# Clinical and Microbiological Efficacy of the Antibacterial Drug Fluorothiazinone in the Treatment of Recurrent Cystitis in a Randomized Clinical Trial

**DOI:** 10.3390/antibiotics15101029

**Published:** 2026-10-16

**Authors:** Nailya A. Zigangirova, Dmitry Yu. Pushkar, Nataliya E. Bondareva, Nadezda L. Lubenec, Tamara S. Perepanova, Elena A. Smolyarchuk, Vladimir B. Beloborodov, Andrey V. Zaycev, Sergey K. Zyryanov, Maria A. Kareva, Evgenia U. Levchenko, Anna B. Sheremet, Alexander L. Gintzburg

**Affiliations:** 1 National Research Center for Epidemiology and Microbiology Named After the Honorary Academician N. F. Gamaleya, 18 Gamaleya St., 123098 Moscow, Russia; 2 Federal State Budgetary Institution of Higher Education “Russian University of Medicine” Ministry of Health of Russia, 4 Dolgorukovskaya St., 127006 Moscow, Russia; 3 State Budgetary Healthcare Institution of the City of Moscow, the S.P. Botkin State Clinical Hospital of the Moscow Department of Health, 2nd Botkin Passage, 5, Building 20, 125284 Moscow, Russia; 4 N.A. Lopatkin Scientific Research Institute of Urology and Interventional Radiology—Branch of the National Medical Research Centre of Radiology of the Ministry of Health of Russian Federation, 3rd Parkovaya Str., 51, 105425 Moscow, Russia; 5 A.P. Nelyubin Institute of Pharmacy of Sechenov First Moscow State Medical University ( Sechenov University), 96k1 Vernadsky Ave., 119571 Moscow, Russia; 6 Department of General and Clinical Pharmacology, Peoples’ Friendship University of Russia Named After Patrice Lumumba (RUDN University), 6 Miklukho-Maklaya St., 117198 Moscow, Russia; 7 ANO “Moscow Center for Innovative Technologies in Healthcare”, 96 Vernadsky Avenue, 119571 Moscow, Russia; 8 Federal State Autonomous Educational Institution of Higher Education I M Sechenov First Moscow State Medical University of the Ministry of Health of the Russian Federation ( Sechenov University), 8/2 Trubetskaya St., 119048 Moscow, Russia

**Keywords:** recurrent cystitis, Fluorothiazinone, nitrofurantoin, clinical trial, non-antibiotic approaches

## Abstract

**Background/Objectives:** New data on the pathogenesis of recurrent urinary tract infections (rUTIs) and the growing level of bacterial resistance to antibiotics require a revision of patient treatment strategies and a search for non-antibiotic approaches to the treatment and prevention of UTIs. The aim of this study was to investigate the clinical and microbiological efficacy of a new, non-traditional antibacterial drug, Fluorothiazinone (FT). **Methods**: We conducted an open-label, randomized, multicenter cohort clinical trial, phase III, at 12 hospitals in Moscow and St. Petersburg. A total of 280 patients were randomized into two groups in a 1:1 ratio to receive FT at a dose of 1200 mg/day for 14 days or furadonin at a dose of 400 mg/day for 7 days. Clinical and microbiological cures were assessed 7, 14, and 28 days after the start of therapy. **Results:** For the primary efficacy endpoint, clinical cure at day 28, the recovery rate was 91.7% in patients receiving FT and 92.8% in patients receiving furadonin, the hypothesis of non-inferiority of FT compared to the standard furadonin regimen was confirmed by two-sided 95% confidence intervals. The proportion of patients with clinical cure on days 7 and 14 also did not differ in the two groups. An assessment of microbiological eradication at all observation times in our study demonstrated that FT, whose mechanism of action is associated with the suppression of pathogen virulence, possesses antibacterial activity comparable to that of the antibiotic furadonin, leading to the eradication of pathogens causing rUTIs. The frequency of microbiological relapses after 28 days of treatment was 19.6% in the group receiving FT and 22.2% in the group receiving furadonin. Both study groups were comparable in the severity of adverse events (AEs). A definite relationship with the study drugs was identified for 1 (11.1%) AE after taking FT and 8 (88.9%) AEs after taking furadonin. **Conclusions:** Clinical trials in patients with recurrent cystitis have demonstrated the intrinsic antibacterial activity of FT, equal clinical and microbiological efficacy compared to first-line drugs for the treatment of recurrent cystitis, as well as a high level of safety, indicating the potential for the use of drugs with a new mechanism of action in the context of growing antibiotic resistance.

## 1. Introduction

Recurrent urinary tract infections (rUTIs) are a significant clinical problem, particularly among women and catheterized patients, leading to diminished quality of life and increased healthcare costs. Recurrent UTIs are defined as two or more episodes of acute bacterial cystitis within six months or at least three episodes within a year [1]. Clinical diagnosis of rUTIs is based on the acute onset of symptoms of dysuria, urinary urgency, urinary frequency, or suprapubic pain. The primary laboratory diagnostic test is the detection of more than 10^5^ colony-forming units (CFU/mL) in urine [2,3]. The causative agents of more than 70% of rUTIs are *Escherichia coli*, while *Klebsiella spp*, *Staphylococcus saprophyticus*, *Proteus mirabilis*, *Enterococcus faecalis*, and other bacteria are detected in a smaller percentage [3,4,5].

Treatment of urinary tract infections (UTIs) is becoming increasingly challenging due to the rapid growth of antimicrobial resistance in bacteria that cause UTIs, particularly in patients with recurrent infections. Nitrofurantoin and fosfomycin are used as first-line therapy for recurrent cystitis [6,7,8,9].

Nitrofurantoin, a broad-spectrum antibiotic, is used to treat UTIs because it typically retains activity against drug-resistant uropathogens [9]. In recent years, two large studies of the etiologic structure and antibiotic susceptibility of cystitis pathogens have been conducted in the Russian Federation, which confirmed the high etiologic significance of *E. coli* (75%) as a causative agent of cystitis and low resistance (<5%) to NF [10]. Therefore, NF remains the drug of choice for empirical therapy according to Clinical Guidelines [11]. The high efficacy of NF in the treatment of rUTI makes it the optimal comparator drug in clinical trials of antimicrobial drugs in patients with cystitis.

The concept of rUTI has changed due to new data on the pathophysiology of rUTI, awareness of the side effects of repeated use of antimicrobials, and the increasing level of bacterial resistance to antimicrobials, which requires a revision of patient treatment strategies [2,12,13,14].

A central factor in the pathogenesis of rUTI is bacterial persistence despite seemingly adequate antibiotic therapy. Unlike classic antibiotic resistance, persistence involves phenotypic adaptations that allow uropathogens to evade both the body’s immune response and antimicrobial agents [15]. The presence of intracellular bacterial communities (ICBCs) in urothelial cells necessitates the use of intracellularly active antimicrobials to suppress not only bacteria present in the urine but also those within the bladder mucosa [3,4].

FT is a new antibacterial drug whose mechanism of action differs from that of antibiotics in that it inhibits virulence rather than suppresses bacterial viability. Preclinical studies of FT have demonstrated suppression of the type III secretion system (T3SS), flagellum-associated bacterial motility, biofilm formation, and decreased bacterial cytotoxicity toward immune cells [16,17]. Suppression of virulence by FT in an infected animal leads to blocking of the infection process and eradication of pathogens with the same effectiveness as antibiotics. FT has significant advantages over antibiotics, including its effectiveness against antibiotic-resistant bacteria, suppression of both extracellular and intracellular pathogens, and the absence of a negative effect on normal microflora [18]. A phase III clinical trial in patients with complicated UTIs, comparing the combination of FT with cefepime versus placebo with cefepime, demonstrated a 25% clinical advantage of FT, while the rate of infection recurrence within 90 days of observation was 10 times lower [19]. Based on the results of this study, FT received state registration in 2024 for the treatment of complicated UTIs in combination with cefepime.

According to the WHO classification, FT is classified as a non-traditional antibacterial agent and is the first in its class and the only registered drug with a mechanism of action based on virulence suppression. To further evaluate the clinical and microbiological efficacy of FT as monotherapy, a clinical trial, “An open clinical trial of the safety and efficacy of the drug Fluorothiazinone, 300 mg tablets (Gamaleya Research Institute of Epidemiology and Microbiology, Health Ministry of the Russian Federation) with the participation of adult patients with chronic bacterial cystitis”, was conducted in comparison with nitrofurantoin. This article presents the results of a study of the clinical and microbiological efficacy of FT, which demonstrated non-inferiority to nitrofurantoin in the treatment of recurrent bacterial cystitis.

## 2. Results

Subjects who met the inclusion criteria and did not have non-inclusion criteria were randomized into two groups in a 1:1 ratio: 140 people who received the drug FT and 140 people who received furadonin. This study was completed according to the protocol: 134 patients in the FT group and 123 patients in the furadonin group (Figure S1: Patient flow and population distribution diagram).

When comparing the demographic and physiological parameters of the patient groups at the time of inclusion in this study and those who received FT or furadonin, reliable differences were found only in the concentration of blood glucose and urea (Table 1); however, the parameters of both groups remained within the normal range, and therefore, their difference was not significant.

At the initial examination (screening stage), all patients complained of sharp pain, a burning sensation during urination, a feeling of fullness in the bladder, frequent urination in small amounts, a nagging pain in the lower abdomen, and changes in the appearance of urine. To assess the individual severity of clinical symptoms, they were scored using the VAS, PUF, and ACSS scales (Table 2). According to the scoring, all patients included in this study had pronounced clinical symptoms of cystitis, with no significant differences between the FT and furadonin groups.

Laboratory evaluation of urine samples collected during the screening phase revealed changes in the characteristic of cystitis (Table 3): decreased clarity, and the presence of mucus, protein, and microhematuria. Leukocyturia was observed in almost all patients. Bacteria and squamous epithelium were detected in more than half of the patients. Nitrates formed as a result of the presence of bacteria were detected in 20%. The severity of these changes was similar in patients receiving FT and furadonin.

Upon further examination, 7% of patients with cystitis were found to have concomitant chronic pathology of various organ systems (Table 4), and the prevalence of its detection also turned out to be approximately the same in the compared groups.

Thus, at the time of inclusion in this study, the two groups of patients with cystitis did not differ significantly in terms of the totality of demographic, physiological, and clinical data; severity of the disease; and severity of laboratory changes.

### 2.1. Primary Efficacy Endpoint

This study’s primary efficacy endpoint was the proportion of patients with clinical cure at 28 days after initiation of treatment. The primary endpoint was assessed in 133 patients who received FT and 125 patients who received furadonin. Due to subjective reasons that arose during this study (missing a control visit and lack of samples for laboratory tests), 23 patients dropped out of this study.

Thus, clinical cure was achieved in 91.7% of patients who received FT and in 92.8% of patients who received furadonin (Table 5). The differences in the proportion of patients with clinical cure between the study groups (p_2_ − p_1_) was 0.0107 in favor of furadonin and were not statistically significant (χ^2^ = 0.170, *p* = 0.918)

Statistical analysis of the non-inferiority hypothesis was used. The non-inferiority margin (d) was pre-specified at d = 15% (0.15). The hypotheses tested were:

- H_0_ (null hypothesis): πs ≥ πe + d—(furadonin is superior to FT by 15% or more);
- H_1_ (alternative hypothesis): πs − d < πe (FT is not inferior to furadonin by more than 15%)

where πs is the clinical cure rate in the furadonin group and πe is the clinical cure rate in the FT group.

Non-inferiority was to be declared if the upper bound of the two-sided 95% confidence interval for the difference in proportions (furadonin − FT) excluded the 15% margin in favor of furadonin. The test was performed at a two-sided significance level of α = 0.05.

The difference in proportions was calculated as (p_2_ − p_1_), where p_2_ is the cure rate in the furadonin group and p_1_ is the cure rate in the FT group. Two-sided 95% confidence intervals were computed.

The clinical cure rate at day 28 was:

- FT group: 91.7% (122/133);
- furadonin group: 92.8% (116/125).

The observed difference in proportions (furadonin − FT is 0.0107 (1.07%) in favor of furadonin.

The upper bound of the two-sided 95% confidence interval for the difference in proportions (furadonin − FT) was:

- 0.084 (8.4%) for Wilson CI [20];
- 0.076 (7.6%) for Wald CI [21,22];
- 0.079 for Newcombe CI [23];
- 0.084 for Newcombe CI with continuity correction.

All the upper bounds were below the pre-specified non-inferiority margin of d = 0.15 (15%). Consequently, the null hypothesis H_0_ was rejected at the significance level α = 0.05 (Table S3: Comparison of proportions furadonin—FT in non-inferiority analysis).

Based on the obtained data, the hypothesis of non-inferiority of FT compared to the standard furadonin regimen is confirmed. It has been demonstrated with 95% confidence that FT is inferior to furadonin by no more than 15% in terms of the proportion of patients achieving clinical cure on day 28 of therapy. The difference in efficacy between the drugs is not statistically significant.

The results indicate that FT is at least as effective as furadonin for treating patients with urinary tract infections within the scope of this study.

### 2.2. Secondary Endpoints of the Study

#### 2.2.1. Clinical Cure After 7 and 14 Days of Treatment

The proportion of patients with clinical cure at days 7 and 14 after initiation of treatment in the CE population was examined (Table 6).

Thus, clinical cure was achieved on day 7 in 69.6% of patients receiving FT and in 75.6% of patients receiving furadonin. There were no statistically significant differences between treatment groups in achieving clinical cure (χ^2^ = 1.222, *p* = 0.543).

Clinical cure on day 14 was achieved in 85.3% of patients receiving FT and in 90.8% of patients receiving furadonin. There were also no statistically significant differences between treatment groups in achieving clinical cure (χ^2^ = 2.307, *p* = 0.316).

Thus, the effectiveness of treating exacerbations of chronic bacterial cystitis with FT was proven with 14-day use.

#### 2.2.2. Assessment of Clinical Response at 90 Days (Telephone Contact)

At Visit 5 (telephone contact), patients were assessed for the presence of genitourinary complaints. Three (2.3%) of the 133 patients in the FT group who completed this visit reported complaints. None of the 124 patients in the furadonin group reported complaints at Visit 5.

#### 2.2.3. Microbiological Evaluation

Other secondary efficacy endpoints were the proportion of patients with microbiological cure on days 7 and 14 after the start of therapy and the number of microbiological relapse on day 28. Midstream urine was collected for microbiological testing. Patients were informed about the rules for collecting urine for microbiological testing. The evaluation was performed in the ME population (all patients from the CE population in whom an uropathogen at ≥10^5^ CFU/mL was isolated during screening) in 97 patients receiving FT and 81 patients receiving furadonin (Table 7, Table S1: Microbiological cure at Visit 2: Comparison between groups, Table S2: Microbiological cure at Visit 3: comparison between groups).

The results of the analysis indicate that there are no statistically significant differences between treatment groups in the frequency of achieving microbiological eradication of initial pathogen in urine. The frequency of microbiological relapses after 28 days of treatment was 19.6% in the group receiving FT and 22.2% in the group receiving furadonin (Table S4: Microbiological relapse at Visit 4: comparison between groups).

In total, within 90 days of the start of therapy, a microbiological relapse was recorded in 20 (19.6%) patients in the FT group and in 18 (22.2%) patients in the furadonin group. No statistically significant differences were found between the groups regarding the incidence of recurrence within 90 days of the start of therapy (χ^2^ = 0.064, *p* = 0.800).

A visit to document the occurrence of a recurrence was conducted for only one patient in the FT group. Data on microbiological relapses for the remaining patients were obtained based on microbiological confirmation during Visit 4 to the study center.

An evaluation of eradication rates for individual pathogens showed that FT exhibits antibacterial activity against the main pathogens causing chronic cystitis, with a 14-day course of therapy being most effective. Furthermore, the relapse rate associated with *E. coli* and *K. pneumoniae* on day 28 after the start of therapy did not differ between the FT group and the furadonin group (Table 8). Thus, furadonin and FT demonstrated equal antibacterial efficacy at 7, 14, and 28 days after initiation of therapy.

#### 2.2.4. The Proportion of Patients Switched to Treatment Other than That Specified by the Protocol, in Case of Treatment Failure and the Need to Prescribe Any Other Antibacterial Drug During the Follow-Up Period up to 14 Days

According to the analysis, two patients (1.4%) in the FT group and three patients (2.1%) in the furadonin group required additional therapy prohibited by the protocol.

No statistically significant differences were found between the groups in the proportion of patients transferred to treatments other than those prescribed by the protocol (χ^2^ = 0.204, *p* = 1.000).

#### 2.2.5. The Proportion of Patients Who Completed the Study According to the Protocol (Compliance)

A secondary endpoint in this study was the proportion of patients who completed this study according to the protocol (compliance).

The results of the analysis showed that 134 (95.71%) patients in the FT group and 123 (87.86%) patients in the furadonin group completed this study in accordance with the protocol. FT was statistically superior to furadonin in terms of the proportion of patients completing this study according to the protocol (χ^2^ = 5.732, *p* = 0.017).

#### 2.2.6. Changes in VAS, PUF, and ACSS Scores

Also, the dynamics of symptoms according to the VAS, PUF and ACSS scales were studied as secondary endpoints (Table 9).

The mean change in the VAS score for the symptom intensity “Pain, discomfort, or burning during urination” in the FT and furadonin groups at Visit 2 was −4.582 and −4.951, respectively; at Visit 3, it was −5.485 and −5.593, respectively; and at Visit 4, it was −5.709 and −5.724, respectively. No statistically significant differences were found between treatment groups (all *p* > 0.05).

The average change in the pelvic pain, urgency, and frequency of urination symptom scale (PUF SCALE) score in the FT and furadonin groups at Visit 2 was −11.575 and −13.423, respectively; at Visit 3, it was −14.866 and −15.480, respectively; and at Visit 4, it was −15.784 and −16.203, respectively. Comparison of the dynamics of scale scores at Visits 3 and 4 revealed no statistically significant differences between the study groups (*p* > 0.05), with the change at Visit 2 being significantly more pronounced in the furadonin group (*p* = 0.046).

The mean change in the total Typical score (ACSS), total Differential score (ACSS), and total QoL score (ACSS) at Visit 2 was −7.746, −0.149, and −3.896, respectively, in the FT group and −9.081, −0.073, and −4.309, respectively, in the furadonin group. At Visit 3, the mean change in the total Typical score (ACSS), total Differential score (ACSS), and total QoL score (ACSS) was −9.552, −0.187, and −4.925, respectively, in the FT group and −10.276, −0.089, and −4.927, respectively, in the furadonin group. At Visit 4, the mean change in the total Typical score (ACSS), total Differential score (ACSS), and total QoL score (ACSS) in points was −10.119, −0.201, and −5.164, respectively, in the FT group and −10.528, −0.089, and −5.041, respectively, in the furadonin group.

A comparison of the dynamics of the Typical, Differential, and QoL scores at Visits 2, 3, and 4 revealed no statistically significant differences between the study groups (*p* > 0.05), with the exception of a change in the total Typical score from Visit 2 to Visit 3 (*p* = 0.003), which favored furadonin.

Thus, the assessment of subjective symptom scores at the end of this study did not reveal statistically significant differences between the study groups, despite some superiority of furadonin in the early stages of treatment (Visit 2, day 7). FT was statistically superior to furadonin in terms of the proportion of patients completing this study according to the protocol (χ^2^ = 5.732, *p* = 0.017).

At study inclusion, the mean VAS scores were 5.750 and 5.757, PUF scores 19.286 and 19.147, and ACSS scores 10.529 and 10.824 in the FT and furadonin groups. By Visit 2, 7 days after the start of therapy, the change in intensity scores on the VAS scale was −4.554 and −4.864; on the PUF scale, −11.496 and −12.903; and on the ACSS scale, −7.659 and −8.900 in the FT and furadonin groups. At Visit 3, 14 days after the start of therapy, the change in the intensity score according to the VAS scale was −5.463 and −5.488; according to the PUF scale, −14.845 and −14.809; and according to the ACSS, −9.485 and −9.939 in the FT and furadonin groups. By Visit 4, 28 days after the start of therapy, on the day of assessment of the primary efficacy endpoint, the change in the intensity score according to the VAS scale was −5.712 and −5.661; according to the PUF scale, −15.850 and −15.787; and according to the ACSS, −10.100 and −10.312 in the FT and furadonin groups. There were no statistically significant differences between the treatment groups (*p* = 0.279). Thus, the dynamics of symptom reduction according to the VAS, PUF, and ACSS scales 7, 14, and 28 days after the start of therapy did not differ in the FT and furadonin groups.

#### 2.2.7. Safety

A total of 334 AEs/SAEs were registered in 117 (42.4%) patients during the clinical trial. Of the 334 registered AEs, 294 (88%) were mild, 39 (11.7%) were moderate, and 1 (0.3%) was severe. For 304 (91.0%) AEs, the outcome was recorded as “Resolved/Recovered”; in 1 case (0.3%), the AE resolved with consequences; in 2 cases (0.6%), there was no recovery (forearm fractures); and the outcome of 27 (8.1%) AEs remained unknown. In 305 (91.3%) AEs, no action was taken regarding the study medications. In 2 cases (0.6%), the drug dose was reduced, and discontinuation was performed in 14 (4.2%) cases. The most common AEs were headache and nausea (Table 10), and there were no serious adverse events.

The incidence of AEs associated with study drugs by system organ class is presented in Table S5: Frequency of adverse events related to the study drugs. An analysis of AEs in relation to the study drugs is presented in Table S6: Comparison of the groups regarding the association of AEs with the study drugs. A definite relationship with the study drugs was identified for 1 (11.1%) AE after taking FT and 8 (88.9%) AEs after taking furadonin.

A comparative analysis of AE frequency revealed no statistically significant between-group differences in the proportion of patients with any AE, AE severity, relationship to study medications, treatments for AEs, or treatments for study medications. Thus, FT demonstrated a high level of safety, comparable to first-line treatment for patients with recurrent cystitis.

## 3. Discussion

The aim of this clinical study was to investigate a new clinical approach to the treatment of recurrent urinary tract infections (RUTIs), based on understanding the mechanism of development of such infections associated with bacterial persistence and the possibilities of using alternative methods of antibacterial therapy. As noted in the Updates to Recurrent Uncomplicated Urinary Tract Infections in Women: AUA/CUA/SUFU Guideline, the growing global crisis of multidrug resistance has also renewed the search for non-antibiotic approaches to the treatment and prevention of urinary tract infections [2,13,14]. One such promising treatment option is the development of phage therapy, which shows promising efficacy similar to antibiotics with minimal side effects [12,24].

The main causative agent of urinary tract infections, *Escherichia coli*, is generally considered an extracellular bacterium; however, uropathogen *E. coli* (UPEC) has recently been shown to be a facultative intracellular pathogen [4]. In experimental mouse models, UPEC has been shown to rapidly replicate, forming large inclusion bodies called intracellular bacterial communities (IBCs) within urothelial cells, from which *E. coli* can either escape and invade other urothelial cells or remain dormant for long periods, where the pathogen is protected from the immune system and remains resistant to standard antibiotic therapy [3,4]. These studies have shown that in recurrent UTIs, the infection is not only extracellular in the urine but also intracellular in the urothelial cells, which is the cause of subsequent UTIs. Due to the inability of many antimicrobial agents to penetrate mammalian cells, infections caused by intracellular bacteria are difficult to treat, requiring the use of antibiotics that are active intracellularly.

The design of this randomized clinical trial was to compare the clinical and microbiological efficacy of a new, non-traditional FT drug in the treatment of chronic cystitis in monotherapy and to demonstrate non-inferiority to the first-line antibiotic furadonin.

FT does not have a direct antibacterial effect, but rather suppresses virulence and, due to its pharmacological properties, effectively penetrates lipid membranes and cells, while maintaining its activity. The antibacterial effect of FT on intracellular pathogens has been demonstrated in epithelial cells and macrophages infected with *C. trachomatis*, *S. enterica*, *E. coli*, and *B. cenocepacia* [25,26,27,28]. For the obligate intracellular pathogen *C. trachomatis*, suppression of the type 3 secretion system with FT led to blocks in the development of chlamydial inclusions in McCoy cells, and in a model of chronic ascending infection in mice caused by this pathogen, treatment with FT possessed antibacterial activity in vivo and was able to control *C. trachomatis* (serovar D) vaginal shedding, ascending infection, and inflammation in the upper genital organs in DBA/2 mice [29].

Earlier, we showed that the compound FT suppressed UPEC’s ability to form biofilms and to move using the flagellum, as well as to penetrate into cells. Prophylactic use with subsequent treatment of FT in rodent models led to an improvement in survival and significantly reduced the bacterial load in the organs of the urinary system, thereby inhibiting the development of ascending infection and preventing the development of pathological changes in prostate tissues [30].

Furadonin, which is successfully used in the treatment of chronic cystitis due to its low resistance rate, was chosen as the comparator drug in this clinical trial. A clinical efficacy comparison demonstrated that FT monotherapy has a similar therapeutic effect to the antibiotic furadonin in the treatment of chronic cystitis. For the primary efficacy endpoint, clinical cure at day 28, the recovery rate was 91.7% in patients receiving FT and 92.8% in patients receiving furadonin.

The proportion of patients with clinical cure on days 7 and 14 also did not differ in the two groups and amounted to 69.6% and 85.3% of patients in the FT group, respectively, and 75.6% and 90.8% of patients in the furadonin group, respectively.

There are summary data on the effectiveness of furadonin based on twenty-seven studies in 4807 patients with urinary tract infections [9]. Clinical cure rates were 79–92%, and microbiological eradication rates were 80–92%. These rates were comparable to those obtained in our study. In a more recent publication of the results of two randomized, multicenter phase III trials (EAGLE-2 and EAGLE-3) comparing the new oral chemotherapy drug gepotidacin and nitrofurantoin in the treatment of uncomplicated UTIs in women, a significant proportion of participants reported complete resolution of symptoms by the end of the study (66.5% with gepotidacin; 64.0% with nitrofurantoin), and another reported improvement of 22.1% (gepotidacin) and 25.9% (nitrofurantoin) from baseline [31]. The lower clinical efficacy rates of furadonin compared with those obtained in our study can be explained by differences in the sensitivity of uropathogens, daily dosage, and duration of furadonin use: 100 mg 2 times a day for 5 days and 100 mg 4 times a day for 7 days, respectively.

An assessment of microbiological eradication at all observation times in our study (days 7, 14, and 28) convincingly demonstrated that FT, whose mechanism of action is associated with the suppression of pathogen virulence, possesses intrinsic antibacterial activity comparable to that of the antibiotic furadonin leading to the eradication of pathogens causing chronic urinary tract infections. The frequency of microbiological relapses after 28 days of treatment was 19.6% in the group receiving FT and 22.2% in the group receiving furadonin. Patient follow-up over 90 days showed that a microbiological recurrence was recorded in only one case in the FT group after Visit 4. Thus, it was shown that the drug, the action of which is realized by suppressing virulence, does not lead to relapses after its discontinuation and allows for treatment in monotherapy mode.

Relief of urinary tract symptoms during the treatment of urinary tract infections is fundamental for assessing the effectiveness of therapy and patient well-being [28]. A subjective assessment of symptom scores at the end of this study revealed no statistically significant differences between the study groups, with the exception of a slight superiority of furadonin in the early stages of treatment (Visit 2, day 7). However, in the FT group, fewer patients dropped out of this study due to a higher number of refusals to participate in the furadonin group, and this difference was statistically significant.

Thus, for the first time, evidence was obtained on the equal clinical efficacy of FT and a first-line antimicrobial agent for the treatment of recurrent cystitis, with both drugs having very high clinical and microbiological efficacy.

The safety of FT compared to furadonin was equal. Of the 334 reported AEs, 88% were mild, 11.7% were moderate, and 1 (0.3%) was severe. Both study groups were comparable in the severity of AEs. A definite relationship with the study drugs was identified for 1 (11.1%) AE after taking FT and 8 (88.9%) AEs after taking furadonin. In the overwhelming majority of patients, AEs were mild and did not require medication adjustments. The most common AEs with FT were headache and nausea. No statistically significant differences were found between the groups in the proportion of patients transferred to treatment other than that prescribed by the protocol.

This study has several limitations. Firstly, when patients were enrolled, they were not stratified by risk factors for chronic cystitis, such as menopause, active sexual activity, urinary tract anomalies, and diabetes mellitus, which significantly impact the effectiveness of antibacterial therapy [2]. This is because the primary objective of this study was to evaluate the therapeutic efficacy of a new class of antivirulence drug used as monotherapy.

In this study, 28-day follow-up after initiation of therapy revealed no advantages of this new approach to treating chronic infections associated with intracellular pathogen persistence. Perhaps, evaluating the results of long-term patient observation will help identify opportunities for more effective treatment of rUTIs and the prevention of relapses using FT. This is the aim of the current clinical trial and subsequent studies.

## 4. Materials and Methods

### 4.1. Study Design

An open-label, randomized, multicenter cohort clinical trial was conducted to investigate the safety and efficacy of the drug FT in antibacterial monotherapy mode compared with furadonin (nitrofurantoin) in patients with chronic bacterial cystitis. This study was conducted in 12 clinical centers in the Russian Federation in 2025–2026 (Table S7: List of research centers and principal investigators). This study was conducted in accordance with study authorization No. 513 dated 30 October 2024 (Ministry of Health of the Russian Federation) and amendment “No. 4310270-25-2/PP” dated 13 January 2025. The study protocol was reviewed and approved by the Ethics Committee of the Ministry of Health of the Russian Federation and the ethics committee of each research center. Written informed consent was obtained from each patient before inclusion in this study.

After enrollment, patients were divided into 2 groups:

1. Cohort of patients (140 people) who received the drug FT;
2. Comparison cohort (140 people) who received the drug furadonin.

The total study duration for each participant was no more than 96 days, including the screening period.

**Figure 1:**
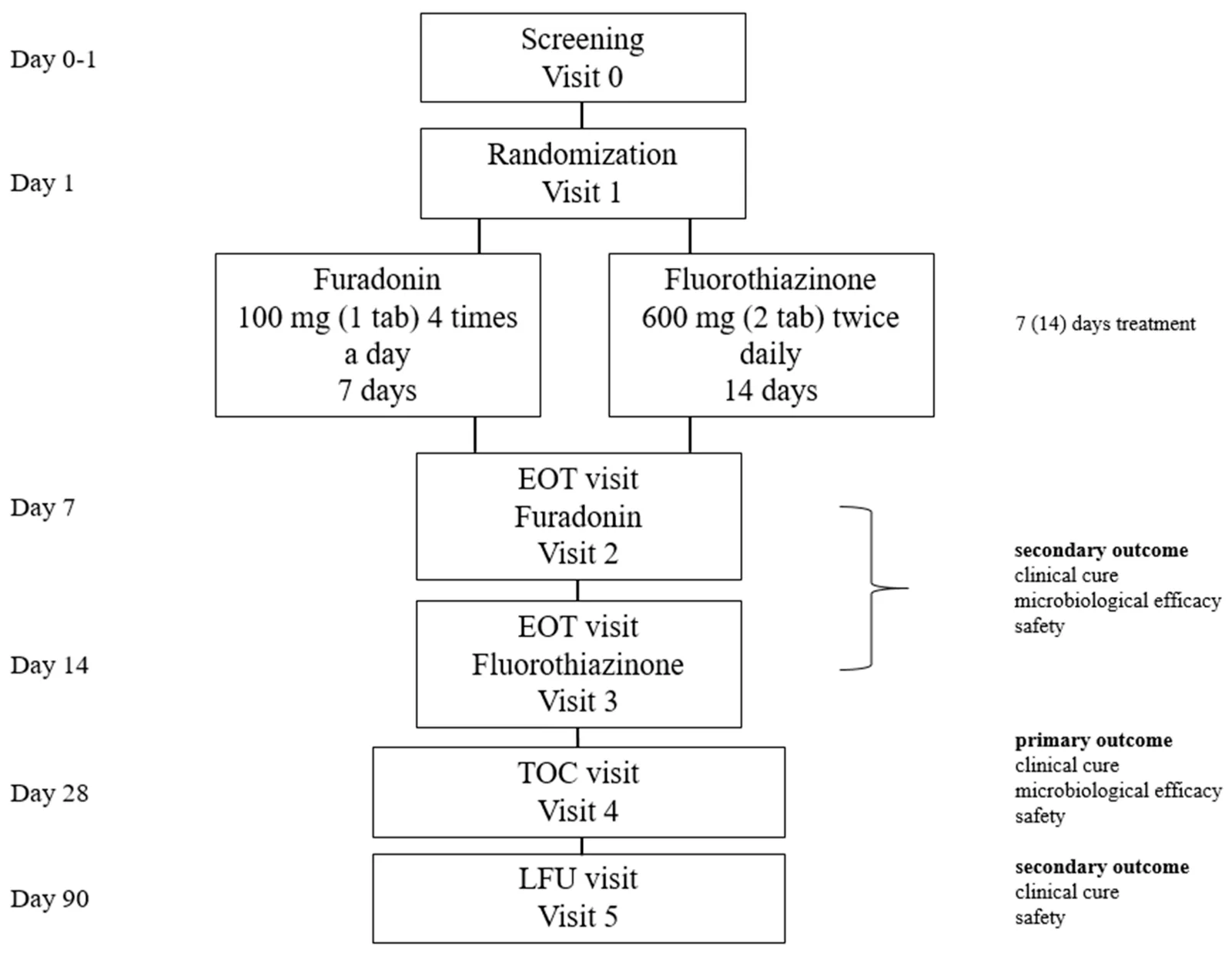
Study design. EOT (end-of-therapy) visit, TOC (test-of-cure) visit, LFU (late-follow-up) visit.

- Screening period (visit 0, day 0–1)—no more than 24 h before the start of treatment;
- Visit 1—start of FT/furadonin treatment;
- Visit 2 EOT (end-of-therapy) visit for furadonin group—7 days of FT/furadonin treatment;
- Visit 3 EOT (end-of-therapy) visit for FT group—14 days of FT treatment;
- Visit 4 TOC (test-of-cure)—28 days after the start of treatment;
- Visit 5—LFU (late-follow-up)—90 days after the start of treatment (Figure 1).

### 4.2. Study Inclusion Criteria

This study enrolled women over 18 years of age with a diagnosis of bacterial cystitis and a history of chronic recurrent bladder infection, as well as characteristic symptoms of an exacerbation of chronic cystitis at the time of enrollment: pain syndrome (pain in the lower abdomen and during urination); dysuria symptoms (frequent urination, pain or burning with urination, imperative urge to urinate, and feeling of incomplete bladder emptying). On the ACSS, the symptom score had to be 6 points or higher. Inclusion criteria also included the patient being treated on an outpatient basis and having no indications for emergency hospitalization at the time of enrollment, as well as the ability to understand the requirements for study participants and willingness to perform the procedures stipulated in the study protocol.

### 4.3. Study Drug Regimen

Patients were randomized into two groups in a 1:1 ratio to receive FT at a dose of 1200 mg/day or furadonin at a dose of 400 mg/day. FT was taken daily, twice a day (2 tablets in the morning and 2 tablets in the evening), for 14 days. Furadonin was taken daily as one tablet, 4 times a day, for 7 days.

### 4.4. Evaluation of Study Results

#### 4.4.1. Analysis Populations

Efficacy and safety were assessed according to the study groups. The following patient populations were studied:

ITT Population—all patients randomized to treatment, regardless of whether they actually received the study drug.

MITT (Modified Intent-to-Treat) Population—all patients in the ITT population who received at least one dose of the study drug.

Clinically Evaluable (CE) Population included patients who met the MITT criteria and met the evaluability criteria (satisfied the main inclusion criteria, had no exclusion criteria, received ≥ 80% of the intended doses, and had no other factors that could interfere with the efficacy assessment).

Microbiologically Evaluable (ME) Population included patients who met the CE criteria and had a properly collected urine sample with an evaluable culture result at screening, and at both the EOT and day 28 (Visit 4) visits.

#### 4.4.2. Efficacy Assessment

##### Clinical Response

At Visits 2 and 3, as well as at the Early Termination Visit (ETV) in case of premature study discontinuation, based on the assessment of signs and symptoms, the investigator selected one of the following clinical outcomes:

Clinical cure—complete resolution or improvement of the signs and symptoms of cystitis that were present at baseline, and the absence of new symptoms requiring antimicrobial therapy outside this study.

Clinical failure—symptoms of cystitis present at enrollment did not completely resolve, new symptoms developed requiring initiation of antibiotic therapy outside this study, or death occurred.

Clinically indeterminate outcome—insufficient data (lack of necessary laboratory and instrumental test results, and failure to attend the visit) to determine cure or failure in the patient.

At Visit 4, based on the assessment of signs and symptoms, the investigator selected one of the following clinical outcomes:

Clinical cure—meeting the criteria for clinical cure and absence of signs of cystitis on day 28.

Relapse—patient met the criteria for clinical cure at Visits 2 or 3 but subsequently developed new signs and symptoms of cystitis by Visit 4 or at an unscheduled visit after day 14, requiring the patient to receive antibiotic therapy for cystitis.

Clinically indeterminate outcome—insufficient data (including lack of necessary laboratory and instrumental test results, and failure to attend the visit) to determine cure or failure in the patient.

If the patient sought medical help for cystitis before Visit 4, unscheduled visit procedures were performed, and antibacterial therapy was prescribed only after these assessments, if necessary.

##### Microbiological Outcome

The microbiological outcome for each patient was determined based on the results of urine cultures at Visits 2 and 3, as well as at the ETV:

Microbiological eradication—all baseline pathogenic microorganisms decreased to <10^4^ CFU/mL in urine culture.

Microbiological persistence—urine culture grew ≥10^4^ CFU/mL of any of the baseline pathogenic microorganisms identified at enrollment.

Microbiologically indeterminate outcome—no urine culture available, the follow-up urine culture cannot be interpreted for any reason, or the follow-up urine culture is considered contaminated.

The microbiological outcome for each patient was determined based on the results of urine cultures as one of the following outcomes at Visit 4, as well as at the RR visit:

Microbiological eradication—all baseline pathogenic microorganisms decreased to <10^4^ CFU/mL in urine culture.

Microbiological relapse—isolation of baseline pathogenic microorganisms at a titer of ≥10^4^ CFU/mL in urine culture.

Microbiologically indeterminate outcome—no urine culture available, or the follow-up urine culture cannot be interpreted for any reason, or the follow-up urine culture is considered contaminated.

#### 4.4.3. Efficacy Evaluation Criteria

The primary efficacy endpoint in this clinical study was the proportion of patients with clinical cure 28 days after the start of treatment.

Secondary endpoints included:

- The proportions of patients with clinical cure on days 7 and 14;
- The proportions of patients with microbiological eradication on days 7 and 14;
- The proportions of patients with microbiological relapse on day 28;
- The proportions of patients with microbiological relapse within 90 days;
- The proportion of patients switched to treatment other than that specified by the protocol, in case of treatment failure and the need to prescribe any other antibacterial drug during the follow-up period up to 14 days;
- Change in scores on the VAS, PUF, and ACSS scales;
- The proportion of patients with AEs, SAEs, ARs, and SARs.

### 4.5. Safety Assessment

The safety of the study therapy was assessed by identifying and recording all potential adverse events that occurred in patients during their participation in this study.

### 4.6. Sample Size Calculation

The primary objective of this study was to demonstrate the non-inferiority of FT compared to the standard regimen of furadonin.

The calculation for standard non-inferiority study conditions was performed using the Sealed envelope calculator [https://www.sealedenvelope.com/power/binary-noninferior URL (accessed on 15 November 2024)]. This calculator is designed for binary outcomes in parallel-group non-inferiority trials comparing an investigational drug to standard treatment.

According to the calculated data, if there is truly no difference between standard and experimental treatment (70% in both groups), then including 232 patients should show 80% probability that the upper limit of the one-sided 95% confidence interval (or equivalently, the two-sided 90% confidence interval) will exclude a difference in favor of the standard group of more than 15%.

Considering a potential dropout rate of 20% of patients and rounding the calculated values to tens, a target of 140 patients in each group (280 patients total) was established for enrollment.

#### 4.6.1. Randomization

Randomization of patients was carried out on the day of study drug administration. The randomization day coincided with the first day of drug administration (Visit 1). The randomization scheme was prepared before the start of this study. This study was open-label, meaning both the investigator and the patient were aware of the treatment assignment. This design was chosen as it was considered suitable for the non-inferiority objective, as the primary endpoint (clinical cure) is relatively objective and the risk of bias was deemed low.

#### 4.6.2. Statistical Analysis

Mathematical and statistical analysis of the results was carried out using Microsoft Excel packages with the AtteStat add-in and Statistica 10.0, or similar programs.

Enrollment in this study, protocol deviations, premature discontinuation of study drug, and withdrawal from this study were presented by group. Summary data on demographic characteristics (age, race, ethnicity, and gender), medical and surgical history, baseline assessment of clinical signs and symptoms, microbiological assessment, and study drug administration were also presented.

Statistical processing of safety, tolerability, and efficacy results was carried out in several stages: determination of the analysis population; determination of data type; determination of the distribution type of quantitative data; data presentation (descriptive statistics); and determination of the method for data analysis and comparison.

Descriptive statistics for quantitative data were presented according to the results of the distribution type analysis. In accordance with the recommendations for data that do not follow a normal distribution, the median (Me) was used as a measure of central tendency for quantitative variables, and the upper (UQ) and lower quartiles (LQ) were used as measures of dispersion. For normally distributed data, the arithmetic mean (Mean) was used as a measure of central tendency, and the standard deviation (SD) was used as a measure of dispersion. The minimum (Min) and maximum (Max) values of the indicator were given.

Ordinal, categorical, and qualitative data were presented as absolute frequencies (number of observations), relative frequencies (proportions, %), and 95% confidence intervals (CI) of the proportion.

For the analysis and comparison of quantitative variables, parametric (in case of normal distribution) and non-parametric (in case of rejection of the hypothesis of normal distribution) statistical methods were used.

To compare data not following a normal distribution, non-parametric tests were calculated. To identify significant changes in dependent samples at different study stages at two time points, the paired Wilcoxon test was used; at three or more time points, Friedman’s rank analysis of variance (Friedman test) was used. For intergroup comparison (Group 1 ↔ Group 2), the Mann–Whitney U test was used.

For normally distributed data, Student’s *t*-test for independent samples was calculated for intergroup comparison, and Student’s *t*-test for dependent samples was used to assess differences at two observation points for each group. To assess within-group differences between three or more observation points, Repeated Measures ANOVA was used.

To compare qualitative indicators (proportions) and assess the significance of detected differences in their frequency, Fisher’s exact test or Pearson’s chi-square test (χ^2^ test) was used, depending on the sample size.

All statistical tests were two-sided, and a *p*-value of <0.05 was considered statistically significant.

Concomitant diseases and adverse events were coded using the MedDRA classification. Concomitant therapy was coded using the ATC system.

In addition to frequency indicators, the values of all quantitative safety indicators (physical examination data, vital signs, and laboratory results) will be presented by visit and by group in tabular form.

## 5. Conclusions

The results of a comparative randomized clinical trial of the efficacy of FT and furadonin in patients with recurrent cystitis demonstrated FT’s antibacterial activity, equal clinical and microbiological efficacy compared to the first-line drug for the treatment of recurrent cystitis, and a high level of safety. The obtained results demonstrate the potential of a new approach to the treatment of chronic infections in the context of increasing antibiotic resistance, an approach associated with the use of antibacterial drugs with a new mechanism of action, to which the risk of developing resistance is reduced.

## Figures and Tables

**Table 1:** Demographic, physiological, and laboratory parameters of patients at the time of inclusion in this study.

Indicator	FT (n = 140)	Furadonin (n = 136)	*p*
Age, years	47 ± 1.4	45 ± 1.2	0.133
Weight, kg	70 ± 0.9	71 ± 1.1	0.806
BMI, kg/m^2^	166.9 ± 0.45	166.2 ± 0.45	0.283
Temperature, °C	36.5 ± 0.15	36.5 ± 0.16	0.771
SBP, mmHg	119 ± 9.1	118 ± 8.2	0.542
DBP, mmHg	74 ± 6.7	73 ± 6.6	0.676
HR, bpm	72 ± 5.4	72 ± 5.5	0.184
RR, bpm	16 ± 1.1	16 ± 1.1	0.949
Blood counts			
Hemoglobin, g/L	132 ± 13.2	131 ± 11.3	0.630
Red blood cells, 10^12^/L	4.4 ± 0.39	4.5 ± 0.40	0.524
Hematocrit, %	40 ± 0.36	40 ± 3.43	0.416
Platelets, 10^9^/L	261.34 ± 59.604	271.29 ± 62.877	0.179
Leukocytes, 10^9^/L	6.8 ± 1.88	6.8 ± 1.54	0.975
Glucose, mmol/L	5.0 ± 0.72	4.8 ± 0.79	0.043
Creatinine, µmol/L	70 ± 12.5	71 ± 10.8	0.93
Total bilirubin, µmol/L	9.9 ± 0.47	9.9 ± 0.39	0.88
Total protein, g/L	73.0 ± 0.36	72.3 ± 0.39	0.21
AST, U/L	20 ± 0.7	20 ± 0.6	0.70
ALT, U/L	20 ± 0.7	18 ± 0.8	0.65
Urea, mmol/L	5.0 ± 0.1	4.7 ± 0.1	0.06
Alkaline phosphatase, U/L	70 ± 2.0	66 ± 1.7	0.22
Lactate dehydrogenase, U/L	205 ± 5.1	209 ± 5.7	0.66

**Table 2:** Total score of cystitis symptoms in patients at the time of screening.

Evaluation Method	FT (n = 140)	Furadonin (n = 136)	*p*
“Pain, discomfort, or burning when urinating” on the VAS scale, points	5.750 ± 0.1470	5.757 ± 0.1463	0.972
Pelvic pain, urgency, and frequency urinary symptom scale (PUF SCALE) score, points	19.286 ± 0.6348	19.140 ± 0.6151	0.869
Acute Cystitis Symptom Scale (ACSS) score, Total (Typical) score	10.529 ± 0.2187	10.824 ± 0.2434	0.368

**Table 3:** Absolute values of urine pH and specific gravity, and relative deviations (%) from normal urine values in patients with cystitis at the time of inclusion in this study.

Indicator	FT (n = 140)	Furadonin (n = 136)	*p*
Absolute indicators			
pH	5.7 ± 0.78	5.8 ± 0.79	0.47
Specific gravity, g/L	1017 ± 7.7	1018 ± 7.4	0.32
Relative indicators (number (%) of patients with deviation from N)			
	FT (n = 140)	furadonin (n = 136)	Normal values
Color	2.1%	1.5%	Light yellow
Transparency	25.7%	29.4%	Clear
Mucus	71.4%	76.5%	No
Protein > 0.15 g/L	26.4%	22.8%	<0.15 g/L
Nitrites	20.7%	22.1%	No
Ketones	2.1%	2.9%	No
Urobilinogen	44.3%	47.1%	<17 μmol/L
Bilirubin	3.6%	0.7%	No
Glucose	0.7%	5.1%	No
Leukocyte esterase	52.9%	47.1%	No
Erythrocytes	60.0%	57.4%	No
Leukocytes > 6 in the field of view	91.4%	94.1%	<6 in the field of view
Hyaline casts	7.1%	4.4%	No
Granular casts	10.7%	5.9%	No
Epithelial casts	36.4%	22.8%	No
Squamous epithelium	60.7%	57.4%	<15 in the field of view
Transitional epithelium	9.3%	6.6%	No
Bacteria	53.6%	66.2%	No

**Table 4:** Comorbidities in patients with cystitis identified at inclusion in this study.

Area of Associated Pathology	FT (n = 140)	Furadonin (n = 136)
Skin and visible mucous membranes	2 (1.4)	1 (0.7)
Musculoskeletal system	2 (1.4)	1 (0.7)
Cardiovascular system	1 (0.7)	2 (1.4)
Respiratory system	1 (0.7)	2 (1.4)
ENT organs	1 (0.7)	0
Digestive system	2 (1.4)	1 (0.7)
Central nervous system	1 (0.7)	1 (0.7)
Total	10 (7%)	9 (7%)

**Table 5:** Clinical efficacy between groups of CE population receiving FT or furadonin on day 28 after the start of therapy.

Group	Clinical Effect	Frequency	Percent	Acceptable Percent	Accumulated Percent
FT	Clinically uncertain outcome	8	6.0	6.0	6.0
	Clinical cure	122	91.7	91.7	97.7
	Relapse	3	2.3	2.3	100.0
	Total	133	100.0	100.0	
Furadonin	Clinically uncertain outcome	7	5.6	5.6	5.6
	Clinical cure	116	92.8	92.8	98.4
	Clinical failure	2	1.6	1.6	100.0
	Total	125	100.0	100.0	
Chi-square criteria					
	Meaning	df	Asymptotic significance (2-sided)	Precise significance (2-sided)	Precise significance (1-sided)
Pearson’s chi-square	0.170 ^a^	2	0.918		
Likelihood ratios	0.171	2	0.918		
Number of admissible observations	258				

^a^. For the number of cells 2 (33.3%), the assumed value is less than 5. The minimum expected number is 2.42.

**Table 6:** Clinical efficacy of treatment with FT and furadonin 7 and 14 days after the start of therapy.

**Clinical Efficacy 7 Days After Initiation of Treatment**					
**Group**	**Clinical Effect**	**Frequency**	**Percent**	**Acceptable** **Percent**	**Accumulated** **Percent**
FT	Clinical failure	5	3.6	3.6	3.6
	Clinically uncertain outcome	37	26.8	26.8	30.4
	Clinical cure	96	69.6	69.6	100.0
	Total	138	100.0	100.0	
Furadonin	Clinical failure	4	3.1	3.1	3.1
	Clinically uncertain outcome	28	21.4	21.4	24.4
	Clinical cure	99	75.6	75.6	100.0
	Total	131	100.0	100.0	
Chi-square criteria					
	Meaning	df	Asymptotic significance (2-sided)	Precise significance (2-sided)	Precise significance (1-sided)
Pearson’s chi-square	1.222 ^a^	2	0.543		
Likelihood ratios	1.225	2	0.542		
Number of admissible observations	269				
**Clinical efficacy 14 days after initiation of treatment**					
**Group**	**Clinical effect**	**Frequency**	**Percent**	**Acceptable** **percent**	**Accumulated** **percent**
FT	Clinical failure	6	4.4	4.4	4.4
	Clinically uncertain outcome	14	10.3	10.3	14.7
	Clinical cure	116	85.3	85.3	100.0
	Total	136	100.0	100.0	
Furadonin	Clinical failure	5	3.8	3.8	3.8
	Clinically uncertain outcome	7	5.4	5.4	9.2
	Clinical cure	118	90.8	90.8	100.0
	Total	130	100.0	100.0	
Chi-square criteria					
	Meaning	df	Asymptotic significance (2-sided)	Precise significance (2-sided)	Precise significance (1-sided)
Pearson’s chi-square	2.307 ^b^	2	0.316		
Likelihood ratios	2.351	2	0.309		
Number of admissible observations	266				

^a^. For the number of cells 2 (33.3%), the assumed value is less than 5. The minimum expected number is 4.38. ^b^. For the number of cells 0 (0.0%), the assumed value is less than 5. The minimum expected number is 5.38.

**Table 7:** Microbiological eradication of all pathogens from urine on days 7 and 14, and the number of microbiological relapses on day 28 after the start of treatment.

Effect, Observation Period	FT n (%)	Furadonin n (%)	χ^2^	*p*
Eradication, Day 7	66 (66.7)	59 (71.1)	0.410	0.522
Eradication, Day 14	79 (80.6)	63 (75.9)	0.589	0.443
Microbiological relapse, Day 28	19 (19.6)	18 (22.2)	0.200	0.655

**Table 8:** Microbiological eradication of pathogens from urine on days 7 and 14, and the number of microbiological relapses on day 28 after the start of treatment.

Bacteria	FT n (%)	Furadonin n (%)
Visit 0 (screening)		
*E. coli*	59	50
*K. pneumoniae*	14	14
*E. faecalis*	20	21
Visit 2 (7th day)		
*E. coli*	24 (59.3)	34 (68.0)
*K. pneumoniae*	9 (64.3)	13 (92.9)
*E. faecalis*	14 (70.0)	17 (81.0)
Visit 3 (14th day)		
*E. coli*	42 (71.2)	34 (68.0)
*K. pneumoniae*	11 (78.6)	14 (100.0)
*E. faecalis*	16 (80.0)	20 (95.2)
Visit 4 (28th day)		
*E. coli*	10 (16.9)	8 (16)
*K. pneumoniae*	1 (7.1)	1 (7.1)
*E. faecalis*	7 (35.0)	5 (23.8)

**Table 9:** Assessment of the intensity of the symptom “Pain, discomfort, or burning during urination” according to the VAS, PUF, and ACSS scales.

	Screening		Visit 1		Visit 2		Visit 3		Visit 4	
	FT N = 140	Furadonin N = 136	FT N = 140	Furadonin N = 136	FT N = 138	Furadonin N = 131	FT N = 136	Furadonin N = 130	FT N = 133	Furadonin N = 125
Assessment of the intensity of the symptom “Pain, discomfort, or burning during urination” on the VAS scale										
Average value	5.750	5.757	5.750	5.757	1.196	0.893	0.287	0.269	0.038	0.096
Standard error	0.1470	0.1463	0.1470	0.1463	0.1088	0.1148	0.0684	0.0723	0.0197	0.0501
Standard deviation	1.7388	1.7062	1.7388	1.7062	1.2779	1.3141	0.7973	0.8239	0.2272	0.5597
Pelvic Pain, Urgency, and Frequency (PUF) Symptom Scale (PUF SCALE)										
Average value	19.286	19.147	19.286	19.140	7.790	6.244	4.441	4.338	3.436	3.360
Standard error	0.6348	0.6145	0.6348	0.6151	0.4287	0.3691	0.2843	0.2567	0.1892	0.1861
Standard deviation	7.5114	7.1663	7.5114	7.1732	5.0363	4.2246	3.3150	2.9269	2.1824	2.0806
Acute Cystitis Symptom Scale (ACSS) score										
Average value	10.529	10.824	10.529	10.824	2.870	1.924	1.044	0.885	0.414	0.512
Standard error	0.2187	0.2434	0.2187	0.2434	0.2295	0.2188	0.1610	0.1600	0.0841	0.1253
Standard deviation	2.5879	2.8386	2.5879	2.8386	2.6958	2.5042	1.8772	1.8242	0.9702	1.4006

**Table 10:** Adverse events by groups.

AEs/SAEs	Absolute Value, Number of AEs/SAEs			Relative Importance, Number of AEs/SAEs		
	Total	FT	Furadonin	Total	Ft	Furadonin
Headache [10019211]	58	37	21	17.4	21.3	13.1
Nausea [10028813]	45	10	35	13.5	5.7	21.9
Dizziness [10013573]	28	12	16	8.4	6.9	10.0
Diarrhea [10012735]	22	13	9	6.6	7.5	5.6
Asthenia [10003549]	21	14	7	6.3	8.0	4.4
Decreased appetite [10061428]	20	11	9	6.0	6.3	5.6
Hyperhidrosis [10020642]	12	7	5	3.6	4.0	3.1
Abdominal pain [10000081]	11	3	8	3.3	1.7	5.0
Increased bilirubin levels in the blood [10005364]	9	9	0	2.7	5.2	0.0
Leukocyturia [10050791]	7	4	3	2.1	2.3	1.9
Vomiting [10047700]	6	3	3	1.8	1.7	1.9
Decreased hemoglobin levels [10018884]	6	6	0	1.8	3.4	0.0
Hypersthenuria [10020770]	4	2	2	1.2	1.1	1.3
Increased lactate dehydrogenase levels in the blood [10005630]	4	4	0	1.2	2.3	0.0
Hematuria [10018867]	4	2	2	1.2	1.1	1.3
Chest pain [10008479]	4	2	2	1.2	1.1	1.3
Other	77	35	42	21.7	20.4	23.6
Overall summary	334	174	160	100	100	100

## Data Availability

The provision of data is limited due to the privacy policies of the funding and regulatory organizations. The possibility of providing data can be considered upon request to the corresponding author. Proposals will be reviewed and approved by the sponsor, security department, researcher, and staff on the basis of scientific merit and absence of competing interests. Once the proposal has been approved, data can be transferred through a secure online platform after the signing of a data access agreement and a confidentiality agreement.

## Supplementary Materials

The following supporting information can be downloaded at https://www.mdpi.com/article/10.3390/antibiotics15101029/s1, Figure S1: Patient flow and population distribution diagram; Table S1: Microbiological cure at Visit 2: Comparison between groups; Table S2: Microbiological cure at Visit 3: comparison between groups; Table S3: Comparison of proportions furadonin—FT in non-inferiority analysis; Table S4: Microbiological relapse at Visit 4: comparison between groups; Table S5: Frequency of adverse events related to the study drugs; Table S6: Comparison of the groups regarding the association of AEs with the study drugs; Table S7: List of research centers and principal investigators.
