# Supplementary material for: Clinical and Microbiological Efficacy of the Antibacterial Drug Fluorothiazinone in the Treatment of Recurrent Cystitis in a Randomized Clinical Trial
Source: Antibiotics (Basel). 2026 Oct 16;15(10):1029. doi: 10.3390/antibiotics15101029 (PMC13700838; doi:10.3390/antibiotics15101029)
Supplement: Supplementary file 1 [file antibiotics-15-01029-s001.zip › antibiotics-4576059-supplementary.pdf]

Figure S1: Patient flow and population distribution diagram

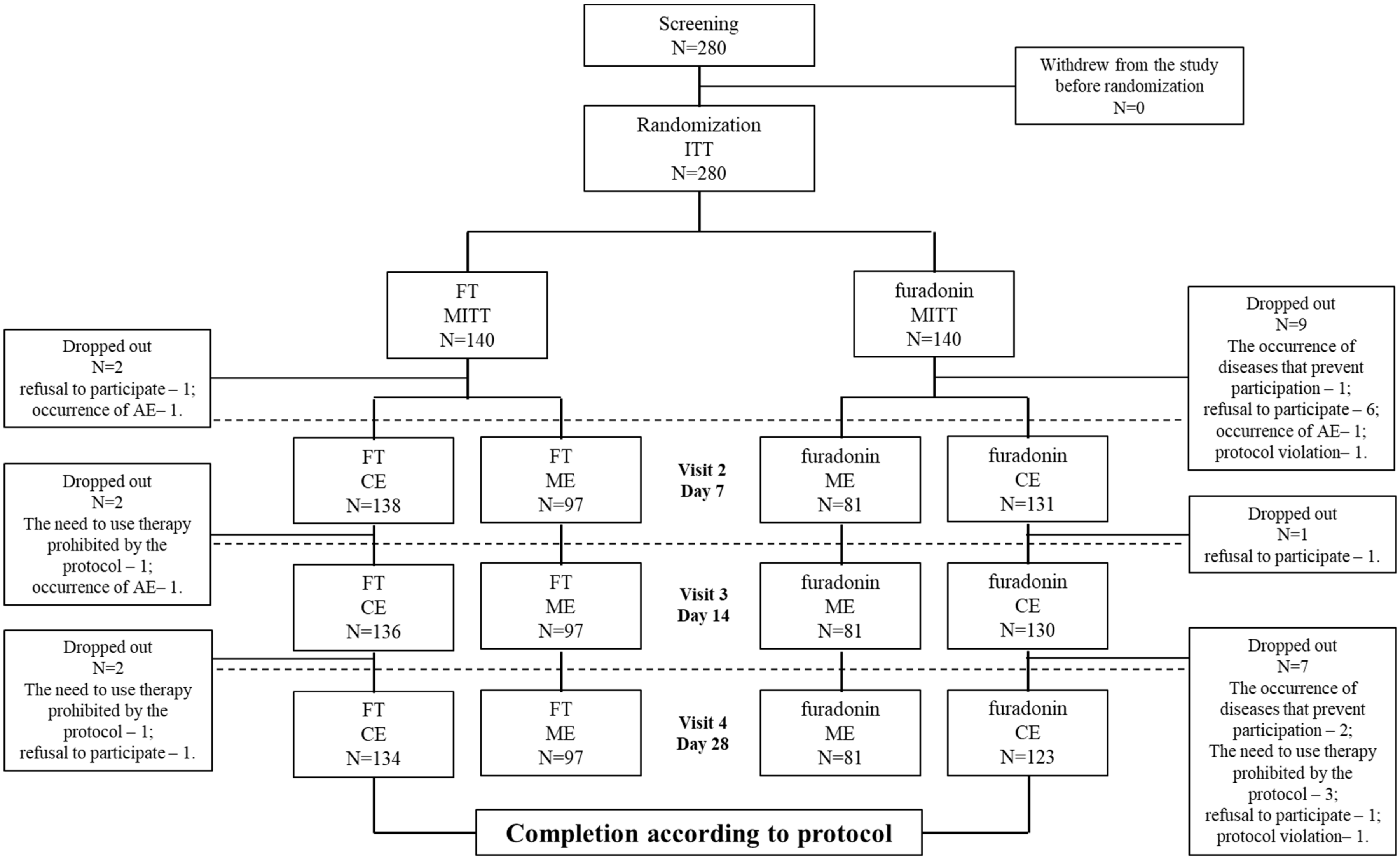

Table S1. Microbiological Cure at Visit 2: Comparison between groups

Quantity				
		Drug		Total
		FT	Furadonin	
Microbiological outcome	persistence	33	24	57
	eradication	66	59	125
Total		99	83	182

Chi-square criteria					
	Value	df	Asymptotic significance (2-tailed)	Exact significance (2-tailed)	Exact significance (1-tailed)
Pearson's chi-square test	.410[a]	1	.522		
Continuity correction[b]	.230	1	.632		
Likelihood ratio	.411	1	.522		
Fisher's exact test				.630	.316
Number of acceptable observations	182				
a. For the number of cells 0 (0.0%) the assumed value is less than 5. The minimum expected number is 25.99.					
b. Calculated only for 2x2 table					

Table S2. Microbiological Cure at Visit 3: Comparison between groups

Quantity				
		Drug		Total
		FT	Furadonin	
Microbiological outcome	persistence	19	20	39
	eradication	79	63	142
Total		98	83	181

Chi-square criteria					
	Value	df	Asymptotic significance (2-tailed)	Exact significance (2-tailed))	Exact significance (1-tailed)
Pearson's chi-square test	.589[a]	1	.443		
Continuity correction[b]	.344	1	.558		
Likelihood ratio	.588	1	.443		
Fisher's exact test				.472	.278
Number of acceptable observations	181				
a. For 0 cells (0.0%), a value less than 5 is assumed. The minimum expected number is 17.88.					
b. Calculated only for 2x2 table					

Table S3: Comparison of proportions furadonin – FT in non-inferiority analysis

Comparison of proportions furadonin - FT	
Nitrofurantoin	0.928 (n=116/125)
FT	0.917 (n=122/133)
Difference	0.011
SE	0.033
95% CI (Wilson)	(-0.062, 0.084)
95% CI (Wald)	(-0.054, 0.076)
95% CI (Newcombe)	(-0.058, 0.079)
95% CI (Newcombe with continuity correction)	(-0.064, 0.084)
Z-statistic[1]	0.09[1]
Chi-square	0.008
df[2]	1[2]
P-value	0.929
[1] *with continuity correction*	
[2] *df = degrees of freedom (for 2×2 table, df = 1)*	
Proportions are n(successes)/n(all)	

Table S4. Microbiological relapse at Visit 4. Comparison between groups

Quantity				
		Drug		Total
		FT	Furadonin	
Microbiological outcome	relapse	19	18	37
	eradication	76	61	137
Total		95	79	174

Chi-square criteria					
	Value	df	Asymptotic significance (2-tailed)	Exact significance (2-tailed)	Exact significance (1-tailed)
Pearson's chi-square test	.200[a]	1	.655		
Continuity correction[b]	.068	1	.794		
Likelihood ratio	.199	1	.655		
Fisher's exact test				.712	.396
Number of acceptable observations	174				
a. Visit = Visit 4					
b. For 0 cells (0.0%), a value less than 5 is assumed. The minimum expected number is 16.80.					
C. Calculated only for 2x2 table					

Table S5. Frequency of adverse events related to the study drugs

Frequency of AEs by connections	Absolute value			Relative value, %		
	FT	Furadonin	Total	FT	Furadonin	Total
**Probable**	7	11	18	4.0	6.9	5.4
**Gastrointestinal disorders [10017947]**	6	9	15	85.7	81.8	83.3
Abdominal pain [10000081]	1	0	1	14.3	0.0	5.6
Diarrhoea [10012735]	4	0	4	57.1	0.0	22.2
Vomiting [10047700]	1	0	1	14.3	0.0	5.6
Nausea [10028813]	0	9	9	0.0	81.8	50.0
**Laboratory and instrumental findings [10022891]**	1	0	1	14.3	0.0	5.6
Decreased hemoglobin concentration [10018884]	1	0	1	14.3	0.0	5.6
**Immune system disorders [10021428]**	0	1	1	0.0	9.1	5.6
Hypersensitivity reaction [10020751]	0	1	1	0.0	9.1	5.6
**Nervous system disorders [10029205]**	0	1	1	0.0	9.1	5.6
Dizziness [10013573]	0	1	1	0.0	9.1	5.6
**Possible**	63	43	106	36.2	26.9	31.7
**Gastrointestinal disorders [10017947]**	13	22	35	20.6	51.2	33.0
Abdominal pain [10000081]	0	2	2	0.0	4.7	1.9
Diarrhoea [10012735]	6	4	10	9.5	9.3	9.4
Vomiting [10047700]	1	1	2	1.6	2.3	1.9
Xerostomia [10013781]	0	1	1	0.0	2.3	0.9
Nausea [10028813]	6	14	20	9.5	32.6	18.9
**Laboratory and instrumental findings [10022891]**	20	1	21	31.7	2.3	19.8
Elevated alanine aminotransferase [10001551]	1	0	1	1.6	0.0	0.9
Hyperbilirubinemia/ elevated bilirubin [10005364]	9	0	9	14.3	0.0	8.5
Hypercreatininemia/ elevated creatinine [10005483]	2	1	3	3.2	2.3	2.8
Elevated Lactate Dehydrogenase (LDH) [10005630]	3	0	3	4.8	0.0	2.8
Elevated urea levels [10005851]	1	0	1	1.6	0.0	0.9
Decreased hemoglobin concentration [10018884]	3	0	3	4.8	0.0	2.8
Decreased red blood cell count/ Erythropenia [10038153]	1	0	1	1.6	0.0	0.9
**Metabolic and nutritional disorders [10027433]**	7	6	13	11.1	14.0	12.3
Decreased appetite [10061428]	7	6	13	11.1	14.0	12.3
**Skin and subcutaneous tissue disorders [10040785]**	0	3	3	0.0	7.0	2.8
Hyperhidrosis [10020642]	0	1	1	0.0	2.3	0.9
Dermatitis [10012431]	0	1	1	0.0	2.3	0.9
Allergic dermatitis [10012434]	0	1	1	0.0	2.3	0.9
**Blood and lymphatic system disorders [10005329]**	1	0	1	1.6	0.0	0.9
Anemia [10002034]	1	0	1	1.6	0.0	0.9
**Nervous system disorders [10029205]**	13	10	23	20.6	23.3	21.7
Headache [10019211]	9	3	12	14.3	7.0	11.3
Dizziness [10013573]	3	6	9	4.8	14.0	8.5
Migraine [10027599]	1	1	2	1.6	2.3	1.9
**Disorders of the liver and biliary tract [10019805]**	2	0	2	3.2	0.0	1.9
Hyperbilirubinemia [10020578]	2	0	2	3.2	0.0	1.9
**Systemic and injection site (local) reactions [10018065]**	7	1	8	11.1	2.3	7.5
Asthenia [10003549]	6	1	7	9.5	2.3	6.6
Chest pain (Thoracic pain) [10008479]	1	0	1	1.6	0.0	0.9

Frequency of AEs by connections	Absolute value			Relative value, %		
	FT	Furadonin	Total	FT	Furadonin	Total
**Not related to the ID (Investigational Drug)**	51	40	91	29.3	25.0	27.2
**Renal disorders and disorders of the urinary tract [10038359]**	1	2	3	2.0	5.0	3.3
Hematuria [10018867]	1	2	3	2.0	5.0	3.3
**Gastrointestinal disorders [10017947]**	7	8	15	13.7	20.0	16.5
Abdominal pain [10000081]	0	3	3	0.0	7.5	3.3
Abdominal bloating [10000060]	1	0	1	2.0	0.0	1.1
Hemorrhoids [10019022]	0	1	1	0.0	2.5	1.1
Diarrhoea [10012735]	2	1	3	3.9	2.5	3.3
Constipation [10010774]	1	0	1	2.0	0.0	1.1
Vomiting [10047700]	0	1	1	0.0	2.5	1.1
Nausea [10028813]	3	2	5	5.9	5.0	5.5
**Infection and infestation [10021881]**	5	5	10	9.8	12.5	11.0
Bacteriuria [10004056]	1	2	3	2.0	5.0	3.3
Respiratory tract infection [10062352]	2	1	3	3.9	2.5	3.3
Laryngitis [10023874]	0	1	1	0.0	2.5	1.1
Cystitis [10011781]	2	1	3	3.9	2.5	3.3
**Laboratory and instrumental findings [10022891]**	2	0	2	3.9	0.0	2.2
Decreased hemoglobin concentration [10018884]	2	0	2	3.9	0.0	2.2
**Metabolic and nutritional disorders [10027433]**	3	0	3	5.9	0.0	3.3
Decreased appetite [10061428]	3	0	3	5.9	0.0	3.3
**Disorders of the respiratory system, thoracic organs and mediastinum [10038738]**	3	4	7	5.9	10.0	7.7
Oropharyngeal pain [10068319]	0	1	1	0.0	2.5	1.1
Cough [10011224]	1	0	1	2.0	0.0	1.1
Rhinorrhea [10039101]	2	3	5	3.9	7.5	5.5
**Skin and subcutaneous tissue disorders [10040785]**	4	1	5	7.8	2.5	5.5
Hyperhidrosis [10020642]	4	0	4	7.8	0.0	4.4
Rash [10037844]	0	1	1	0.0	2.5	1.1
**Musculoskeletal and connective tissue disorders [10028395]**	0	2	2	0.0	5.0	2.2
Protrusion of the intervertebral disc [10050296]	0	1	1	0.0	2.5	1.1
Spinal pain [10072005]	0	1	1	0.0	2.5	1.1
**Nervous system disorders [10029205]**	16	2	18	31.4	5.0	19.8
Headache [10019211]	13	1	14	25.5	2.5	15.4
Dizziness [10013573]	2	1	3	3.9	2.5	3.3
Dysgeusia [10013911]	1	0	1	2.0	0.0	1.1
**Renal disorders and disorders of the urinary tract [10038359]**	4	7	11	7.8	17.5	12.1
Hypersthenuria [10020770]	2	2	4	3.9	5.0	4.4
Leukocyturia [10050791]	2	3	5	3.9	7.5	5.5
Pollakiuria [10036018]	0	1	1	0.0	2.5	1.1
Proteinuria [10037032]	0	1	1	0.0	2.5	1.1
**Vascular disorders [10047065]**	0	1	1	0.0	2.5	1.1
Hyperemia [10020565]	0	1	1	0.0	2.5	1.1
**Systemic and injection site (local) reactions [10018065]**	5	6	11	9.8	15.0	12.1
Asthenia [10003549]	3	5	8	5.9	12.5	8.8
Chest pain (Thoracic pain) [10008479]	1	1	2	2.0	2.5	2.2

Frequency of AEs by connections	Absolute value			Relative value, %		
	FT	Furadonin	Total	FT	Furadonin	Total
Pyrexia [10037660]	1	0	1	2.0	0.0	1.1
**Injuries, intoxications and procedural complications [10022117]**	1	2	3	2.0	5.0	3.3
Foot fracture [10016970]	0	1	1	0.0	2.5	1.1
Fracture of the humerus [10020462]	1	0	1	2.0	0.0	1.1
Ligament sprain [10024453]	0	1	1	0.0	2.5	1.1
**Unclassified**	2	1	3	1.1	0.6	0.9
**Disorders of the respiratory system, thoracic organs and mediastinum [10038738]**	1	0	1	50.0	0.0	33.3
Hypoxia [10021143]	1	0	1	50.0	0.0	33.3
**Nervous system disorders [10029205]**	1	1	2	50.0	100.0	66.7
Headache [10019211]	1	1	2	50.0	100.0	66.7
**Certain**	1	8	9	0.6	5.0	2.7
**Gastrointestinal disorders [10017947]**	0	7	7	0.0	87.5	77.8
Nausea [10028813]	0	7	7	0.0	87.5	77.8
**Laboratory and instrumental findings [10022891]**	1	0	1	100.0	0.0	11.1
Elevated alanine aminotransferase [10001551]	1	0	1	100.0	0.0	11.1
**Nervous system disorders [10029205]**	0	1	1	0.0	12.5	11.1
Headache [10019211]	0	1	1	0.0	12.5	11.1
**Equivocal**	39	50	89	22.4	31.3	26.6
**Renal disorders and disorders of the urinary tract [10038359]**	1	0	1	2.6	0.0	1.1
Hematuria [10018867]	1	0	1	2.6	0.0	1.1
**Gastrointestinal disorders [10017947]**	5	6	11	12.8	12.0	12.4
Abdominal pain [10000081]	2	2	4	5.1	4.0	4.5
Abdominal bloating [10000060]	1	0	1	2.6	0.0	1.1
Diarrhoea [10012735]	1	2	3	2.6	4.0	3.4
Constipation [10010774]	1	0	1	2.6	0.0	1.1
Nausea [10028813]	0	2	2	0.0	4.0	2.2
**Infection and infestation [10021881]**	1	0	1	2.6	0.0	1.1
Respiratory tract infection [10062352]	1	0	1	2.6	0.0	1.1
**Laboratory and instrumental findings [10022891]**	2	1	3	5.1	2.0	3.4
Hyperglycemia/ Elevated blood glucose [10005557]	1	0	1	2.6	0.0	1.1
Elevated Lactate Dehydrogenase (LDH) [10005630]	1	0	1	2.6	0.0	1.1
Tachycardia [10019303]	0	1	1	0.0	2.0	1.1
**Metabolic and nutritional disorders [10027433]**	0	1	1	0.0	2.0	1.1
Decreased appetite [10061428]	0	1	1	0.0	2.0	1.1
**Disorders of the respiratory system, thoracic organs and mediastinum [10038738]**	2	0	2	5.1	0.0	2.2
Cough [10011224]	1	0	1	2.6	0.0	1.1
Rhinorrhea [10039101]	1	0	1	2.6	0.0	1.1
**Immune system disorders [10021428]**	1	3	4	2.6	6.0	4.5
Hypersensitivity reaction [10020751]	1	3	4	2.6	6.0	4.5
**Skin and subcutaneous tissue disorders [10040785]**	2	7	9	5.1	14.0	10.1
Hyperhidrosis [10020642]	2	4	6	5.1	8.0	6.7
Dermatitis [10012431]	0	1	1	0.0	2.0	1.1
Rash [10037844]	0	2	2	0.0	4.0	2.2
**Blood and lymphatic system disorders [10005329]**	2	0	2	5.1	0.0	2.2

Frequency of AEs by connections	Absolute value			Relative value, %		
	FT	Furadonin	Total	FT	Furadonin	Total
Lymphadenitis [10025188]	1	0	1	2.6	0.0	1.1
Lymphocytosis [10025280]	1	0	1	2.6	0.0	1.1
**Musculoskeletal and connective tissue disorders [10028395]**	1	0	1	2.6	0.0	1.1
Pain in the limb [10033425]	1	0	1	2.6	0.0	1.1
**Nervous system disorders [10029205]**	18	24	42	46.2	48.0	47.2
Headache [10019211]	14	15	29	35.9	30.0	32.6
Dizziness [10013573]	4	8	12	10.3	16.0	13.5
Migraine [10027599]	0	1	1	0.0	2.0	1.1
**Eye disorders [10015919]**	0	2	2	0.0	4.0	2.2
Pain in the eye [10015958]	0	2	2	0.0	4.0	2.2
**Renal disorders and disorders of the urinary tract [10038359]**	1	4	5	2.6	8.0	5.6
Dysuria [10013990]	0	3	3	0.0	6.0	3.4
Urinary urgency [10027566]	0	1	1	0.0	2.0	1.1
Leukocyturia [10050791]	1	0	1	2.6	0.0	1.1
**Systemic and injection site (local) reactions [10018065]**	3	2	5	7.7	4.0	5.6
Asthenia [10003549]	3	1	4	7.7	2.0	4.5
Chest pain (Thoracic pain) [10008479]	0	1	1	0.0	2.0	1.1
**Conditional**	11	7	18	6.3	4.4	5.4
**Gastrointestinal disorders [10017947]**	2	5	7	18.2	71.4	38.9
Abdominal pain [10000081]	0	1	1	0.0	14.3	5.6
Diarrhoea [10012735]	0	2	2	0.0	28.6	11.1
Vomiting [10047700]	1	1	2	9.1	14.3	11.1
Nausea [10028813]	1	1	2	9.1	14.3	11.1
**Metabolic and nutritional disorders [10027433]**	1	2	3	9.1	28.6	16.7
Decreased appetite [10061428]	1	2	3	9.1	28.6	16.7
**Skin and subcutaneous tissue disorders [10040785]**	1	0	1	9.1	0.0	5.6
Hyperhidrosis [10020642]	1	0	1	9.1	0.0	5.6
**Nervous system disorders [10029205]**	3	0	3	27.3	0.0	16.7
Dizziness [10013573]	3	0	3	27.3	0.0	16.7
**Renal disorders and disorders of the urinary tract [10038359]**	2	0	2	18.2	0.0	11.1
Dysuria [10013990]	1	0	1	9.1	0.0	5.6
Leukocyturia [10050791]	1	0	1	9.1	0.0	5.6
**Systemic and injection site (local) reactions [10018065]**	2	0	2	18.2	0.0	11.1
Asthenia [10003549]	2	0	2	18.2	0.0	11.1
**Overall total**	**174**	**160**	**334**	**100**	**100**	**100**

Table S6. Comparison of the groups regarding the association of AEs with the study drugs

Cross-tabulation table				
Quantity				
		Drug		Total
		FT	Furadonin	
Association of AEs with the ID	Probable	7	11	18
	Possible	63	43	106
	Not related to the ID (Investigational Drug)	51	40	91
	Unclassified	2	1	3
	Certain	1	8	9
	Equivocal	39	50	89
	Conditional	11	7	18
Total		174	160	334

Chi-square criteria			
	Value	df	Asymptotic significance (2-tailed)
Pearson's chi-square test	13.455[a]	6	.036
Likelihood ratio	14.235	6	.027
Number of acceptable observations	334		
a. For 4 cells (28.6%), a value less than 5 is assumed. The minimum expected number is 1.44.			

Table S7. List of research centers and principal investigators

Research Center, №	Name of the center	Principal investigator
2	State Budgetary healthcare Institution of the city of Moscow "Moscow Multidisciplinary Clinical Center "Kommunarka" of the Department of Health of the City of Moscow", a separate subdivision of SBHI "MMCC "KOMMUNARKA "	D.N. Protsenko
3	State Budgetary Healthcare Institution of Moscow City Clinical Hospital No. 1 named after N.I. Pirogov of the Moscow Department of Health	A.A. Anderzhanova
4	State Budgetary Healthcare Institution of the city of Moscow "V.P. Demikhov City Clinical Hospital of the Moscow Department of Health"	D.A. Bystritsky
6	Saint Petersburg State Budgetary Healthcare Institution "City Multidisciplinary Hospital No. 2"	V.A. Volchkov
7	State Budgetary Healthcare Institution of the city of Moscow "City Clinical Hospital No. 24 of the Department of Health of the City of Moscow"	S. K. Zyryanov

Research Center, №	Name of the center	Principal investigator
8	State Budgetary Healthcare Institution of the city of Moscow "N.V. Sklifosovsky Scientific Research Institute of Emergency Medicine of the Moscow Department of Health"	V.V. Kulabukhov
9	State Budgetary Healthcare Institution of the city of Moscow "V.M. Buyanov City Clinical Hospital of the Moscow Department of Health"	S.V. Borisovskaya
10	State Budgetary Healthcare Institution of the city of Moscow "I.V. Davydovsky City Clinical Hospital of the Moscow Department of Health"	E. Y. Vasilyeva
11	State Budgetary healthcare Institution of the city of Moscow "V.V. Vinogradov City Clinical Hospital of the Department of Health of the City of Moscow"	Z. D. Kobalava
12	The State Budgetary healthcare Institution of the city of Moscow "V.V. Veresaev Municipal Clinical Hospital of the Moscow Department of Health"	Z.S. Shogenov
13	St. Petersburg State Healthcare Institution "City Hospital No. 15"	B. M. Goloshchekin
16	Federal State Autonomous Educational Institution of Higher Education I.M. Sechenov First Moscow University of the Ministry of Health of the Russian Federation (Sechenov University)	A.G. Yavorovsky
17	Federal State Budgetary Scientific Institution "Russian Scientific Center of Surgery named after Academician B. V. Petrovsky"	D.E. Rozhkov
18	State Budgetary healthcare Institution of the city of Moscow "City Clinical Hospital No. 67 named after L.A. Vorokhobov of the Department of Health of the City of Moscow"	S.Y. Bastrikin
